# A functional shunt in the umbilical cord: the role of coiling in solute and heat transfer

**DOI:** 10.1098/rsif.2025.0148

**Published:** 2025-09-24

**Authors:** Tianran Wan, Edward D. Johnstone, Shier Nee Saw, Oliver E. Jensen, Igor L. Chernyavsky

**Affiliations:** ^1^ Department of Mathematics, University of Manchester, Manchester M13 9PL, UK; ^2^ Maternal and Fetal Health Research Centre, University of Manchester, Manchester M13 9WL, UK; ^3^ Department of Artificial Intelligence, Faculty of Computer Science and Information Technology, Universiti Malaya, Kuala Lumpur 50603, Malaysia

**Keywords:** umbilical cord, vascular configuration, helicity, oxygenation, diffusive solute transport, heat exchange

## Abstract

The umbilical cord plays a critical role in delivering nutrients and oxygen from the placenta to the fetus through the umbilical vein, while the two umbilical arteries carry deoxygenated blood with waste products back to the placenta. Although solute exchange in the placenta has been extensively studied, exchange within the cord tissue has not been investigated. Here, we explore the hypothesis that the coiled structure of the umbilical cord could strengthen diffusive coupling between the arteries and the vein, resulting in a functional shunt. We calculate the diffusion of solutes, such as oxygen, and heat in the umbilical cord to quantify how this shunt is affected by vascular configuration within the cord. We demonstrate that the shunt is enhanced by coiling and vessel proximity. Furthermore, our model predicts that typical vascular configurations of the human cord tend to minimize shunting, which could otherwise disrupt thermal regulation of the fetus. We also show that the exchange, amplified by coiling, can provide additional oxygen supply to the cord tissue surrounding the umbilical vessels.

## 1. Introduction

During pregnancy, fetal development depends on the umbilical cord for delivering nutrients and oxygen from the placenta to the fetus through the umbilical vein (UV) and transferring waste products back through the umbilical arteries (UAs), before they are discarded through the maternal circulatory system. Because the umbilical cord plays such a vital role during pregnancy, disruption to blood flow or abnormality in the structure of the cord could significantly affect the growth of the fetus [1,2].

**Figure 1.**
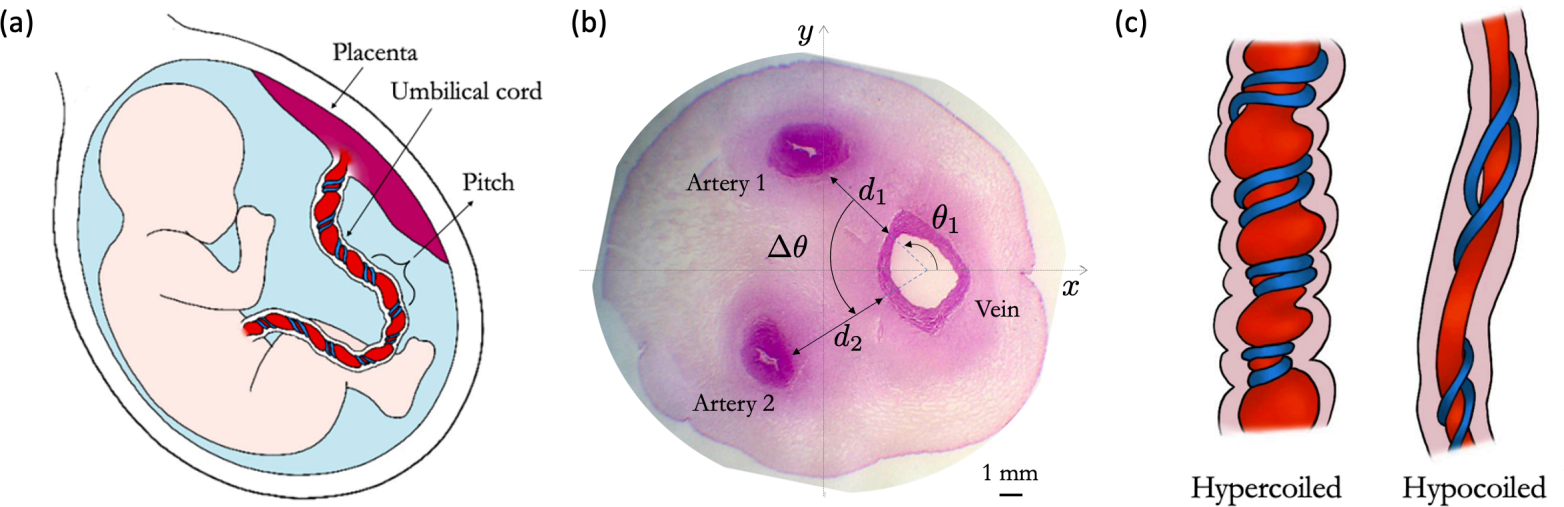
The vascular structure of the umbilical cord. (a) A schematic of the helical structure of the umbilical cord that connects the fetus to the placenta; the pitch 2π/Ω represents the average length of a coil relative to the radius of the cord; Ω is the dimensionless helicity parameter (proportional to the UCI). (b) A histological image of the cross-section of a healthy human cord (adapted from [3] under CC BY-NC 4.0); the vascular cord configuration is characterized by the angle between the UV and the first artery ($\theta_{1}$, measured relative to the x-axis that passes through the centre of the cord and the centre of the vein), the angle between the two arteries Δθ and the artery–vein separation distances $d_{1},\;d_{2}$ normalized by the cord radius. (c) Schematic illustrations of hypercoiled and hypocoiled umbilical cords.

The umbilical vessels are characterized by their helical geometry (figure 1). The umbilical coiling index (UCI), defined as the ratio of the number of coils to the length of the cord, or the inverse helical pitch (figure 1a), is commonly used in clinical practice to quantify the coiled geometry [4]. In normal pregnancies, the mean UCI is approximately 0.17 coils per cm [5]; cords with a UCI below the 10th centile (*ca* 0.07 coils per cm) are classified as hypocoiled, while cords with a UCI above the 90th centile (*ca* 0.3 coils per cm) are classified as hypercoiled (figure 1c). Hypercoiling is associated with an increased incidence of fetal growth restriction (FGR), fetal heart deceleration during delivery, vascular thrombosis and cord stenosis [6]. Hypocoiling is associated with a higher incidence of fetal demise, fetal distress at delivery and chromosomal abnormalities [7].

Given the association between abnormal coiling and adverse pregnancy outcomes, it is important to explore how features of helical cord structure, such as coiling and vascular configuration (figure 1b), may affect umbilical cord function. In the umbilical cord, many solutes come from the placenta, where exchange between fetal and maternal blood takes place. While placental exchange has been widely investigated [8–11] and a few studies have analysed blood flow in umbilical vessels [12–15], to the best of our knowledge, solute exchange between the umbilical vessels remains unexplored.

In addition to its role in solute transport, the cord also contributes to fetal thermoregulation, which is immature compared with adult physiology, as the fetus lacks the ability to sweat or shiver. Instead, the fetus relies on heat exchange through the feto-placental circulation to maintain an appropriate temperature. Extreme heat exposure or maternal hyperthermia during pregnancy can overwhelm the fetus’s limited ability to regulate its own temperature, potentially leading to complications such as preterm birth, low birth weight and congenital anomalies [16]. A recent computational study by Kasiteropoulou *et al.* [17] indicated the importance of umbilical coiling in thermal regulation; however, the overall role of cord structure in feto-maternal heat exchange remains an open question.

Finally, in humans, the cord’s connective tissue, known as Wharton’s jelly (WJ), has no extra vasculature besides the umbilical vessels [18]. In other mammals, several smaller vessels have been found through histological studies [19]. It has been hypothesized that they may act as *vasa vasorum*, smaller vessels that supply the walls of larger blood vessels. Given the coiled nature of the human umbilical vessels, an enhanced exchange could also affect oxygen availability for vessel walls and cells in the extravascular cord tissue.

The objective of this study is therefore to quantify the diffusive coupling of umbilical vessels in the human cord for different solutes and heat by combining a theoretical model with anatomical data. Our model moves beyond the coiling index by encapsulating the complexity of three-dimensional helical vascular configurations in a computationally efficient and interpretable manner, allowing us to define key geometric determinants of functional shunting in the cord for a wide range of solutes. We further contextualize our results for specific structural features of human umbilical cords, based on *ex vivo* histology and *in vivo* ultrasound imaging, and show how typically observed vascular configurations tend to minimize heat exchange between the cord’s vessels. Our model also predicts that cord helicity can enhance oxygen availability to the cord tissue surrounding the umbilical vessels, particularly in the presence of tissue metabolism. The developed methodology provides a building block for future fetal–placental–maternal models, enabling more direct hypothesis testing of structure–function relationships in obstetric pathophysiology, as well as in other complex exchange organs.

## 2. Methods

Our integrative approach is outlined below, starting with a theoretical model that seeks to capture the dominant features of intervascular solute exchange in a coiled umbilical cord.

### 2.1. Modelling solute exchange between helical vessels

We model the umbilical cord as a straight circular cylinder that hosts three helical vessels (one vein and two arteries). Each vessel has a circular cross-section in the plane orthogonal to the axis z of the cylinder and a constant (and common) pitch 2π/Ω, defined as the length of one complete helical turn parallel to the z-axis of the cord, measured relative to the cord’s radius (figure 1a). The dimensionless helicity parameter Ω is proportional to the UCI. The vascular configuration in the cross-section of a cord (figure 1b) can be characterized by the relative distance d between the UA and the vein (scaled by the cord’s radius) and the angles between the vein and each of the two arteries $\theta_{1}$ and $\theta_{2}$ (measured with respect to an axis connecting the centre of the cord to the centre of the vein). For simplicity, we assume that both UAs are of the same size and are the same distance d from the vein (so that $d_{1}=d_{2}=d$ in figure 1b).

We model the transfer of solutes (such as oxygen) and heat in the cord tissue $I_{\text{t}}$ by a steady diffusion-uptake process and assume the cross-sectional solute concentration to be approximately uniform in each umbilical vessel. While blood flow in the UV is approximately steady, with weak fluctuations [20], arterial flow is pulsatile [12]. However, the diffusion (with tissue diffusivity $D_{t}^{*}$) of oxygen and heat between the vessels is much slower ($(d^{*}{)}^{2}/D_{t}^{*}∼$ 10–10^3^ s, for $d^{*}∼1$ mm; table 1 and electronic supplementary material) compared with the typical fetal heart-rate timescale (10^−1^–1 s), justifying the steady-transport approximation. We also neglect transmural flow in the cord tissue [25] and possible differences in solute concentration between the umbilical arterial blood and the amniotic fluid. The governing equations (see electronic supplementary material, S1 for more details) in dimensionless variables describing solute transport in the umbilical tissue are given by

**Table 1.** Key geometric parameters of the model. Stars denote dimensional quantities. The pitch was calculated as the inverse UCI [21], and the artery–vein distance was estimated as described in the electronic supplementary material, S6.

parameter	notation	value (cm)	source
arterial radius	$r_{a}^{*}$	0.06−0.2	[22]
venous radius	$r_{v}^{*}$	0.1−0.4	[22]
cord radius	$R_{c}^{*}$	0.2−1 ^a^	[23]
venous helical radius	$R_{v}^{*}$	0−0.8	[14]
venous wall thickness	$h_{v}^{*}$	0.03−0.05	[24]
arterial wall thickness	$h_{a}^{*}$	0.05−0.08	[24]
cord pitch	$2\pi /\Omega^{*}$	3−14	[21]
cord length	$L^{*}$	50−100	[18]
artery–vein distance	$d^{*}$	0.04−0.4	estimated

^a^ All the dimensionless lengths in the model are scaled on $R_{c}^{*}=1$ cm.


(2.1a)∇2C=α(C+1/β)in It,(2.1b)ηC+(n^⋅∇C)=0on Ic,(2.1c)n^⋅∇C=0on If,\,p,(2.1d)C=0on Ia ifor i=1,2,andC=1on Iv.

Here, C(x,y,z) is the dimensionless solute concentration (or temperature) normalized by the vein–artery concentration difference $C_{v}-C_{a}$ and centred at the arterial concentration $C_{a}$, so that the dimensional concentration is $C_{a}+(C_{v}-C_{a})C$. In (2.1a), α is a dimensionless metabolic rate parameter (assumed zero for all the solutes but oxygen), and $\beta =(C_{v}-C_{a})/C_{a}$ is the relative solute concentration difference between the UA and the UV (electronic supplementary material, S1). Exchange at the interface $I_{\text{c}}$ between the umbilical cord and the amniotic fluid is characterized by the amniotic exchange parameter η in (2.1b); when η=0, no solute is lost through outer cord’s surface; η→∞ in (2.1b) enforces C=0 at the cord–amniotic interface. We impose no-flux conditions in (2.1c) at the tissue cross-sections bounding the fetal and placental ends of the umbilical cord, respectively ($I_{\text{f,}\;\text{p}}$); here, $\hat{n}$ denotes a unit normal pointing outside the domain occupied by the cord tissue $I_{\text{t}}$. Finally, assuming zero axial concentration gradient along the cord, we set fixed solute concentrations on the surface of the UAs $I_{\text{a}{,}_{1}}$, $I_{\text{a}{,}_{2}}$ and the vein $I_{\text{v}}$ in (2.1d), so that we can focus attention on intervascular exchange through cord tissue, accounting for its internal helical structure.

We reduce the three-dimensional problem (2.1) to two dimensions by exploiting the helical coordinate transformation X=xcos⁡(Ωz)+ysin⁡(Ωz), Y=−xsin⁡(Ωz)+ycos⁡(Ωz), Z=z, where z measures the distance along the cord’s axis (see electronic supplementary material, S1 for more details). This transformation allows us to study the three-dimensional effects of helicity in the cross-sectional plane of the cord by solving for C(X,Y), the field that is invariant along the length of the cord relative to a rotating coordinate system. We assume that all vessels share the same pitch and that each vessel centreline maintains a fixed distance from the (straight) cord centreline. The dimensionless solute flux N per unit length of the cord delivered from the UV to the two UAs and cord tissue, and the solute flux per unit length taken up by cord tissue are then given, respectively, by


(2.2) $$N(\Omega ,\alpha ,\beta ,\eta )=\int_{\partial I_{\text{v}}\;\cap \;P}\hat{n}\cdot \left(\nabla_{⊥}C+\Omega^{2}H\right)ds\;\;\text{and}\;\;N_{\text{u}}=\int_{I_{\text{t}}\;\cap \;P}\;\alpha \left(C+1/\beta \right)\;dA\;,$$

where $\nabla_{⟂}\equiv \left[\partial_{X},\;\partial_{Y},\;0\right]$ is the two-dimensional gradient operator, and $H=\left(Y\;C_{X}-X\;C_{Y})\;[Y,\;-X,\;0\right]$ is a flux component arising due to cord helicity (see electronic supplementary material, S2 for further details). The integrals in (2.2) are taken in a plane P, in which z=constant. The solute flux N(Ω,α,β,η) in transformed coordinates shows explicitly the rotational effects of helicity. The helicity factor $\Omega^{2}$ controls the strength of the rotational effects, while H represents how the concentration gradients twist in space. This term shows that concentration not only diffuses radially but also experiences angular distortions relative to the vessels. We explore below the effects of helicity, vessel proximity and the configuration of the vessels within the cord on the solute fluxes. The ratio of a dimensional exchange flux between the umbilical vessels, based on N, and the advective flux along the umbilical cord defines a dimensionless Damköhler number


(2.3) $$\begin{array}{lcr} & \mathrm{Da}=\frac{D_{t}^{*}L^{*}\;N(\Omega ,\alpha ,\beta ,\eta )}{B\;Q^{*}}\;, & \end{array}$$

where $D_{t}^{*}$ is the solute diffusivity in tissue, $L^{*}$ is the cord length, $Q^{*}$ is the total umbilical flow rate and B is an advection-facilitation parameter that accounts for haemoglobin binding of certain solutes, such as oxygen (see electronic supplementary material, S8 and table S3 for more details and estimates of Da for oxygen and heat exchange).

As the metabolic rate α increases, the region surrounding the vessels’ lumens exhibits thin boundary layers and steep concentration gradients. We exclude the effects of these highly localized boundary layers by calculating the extravascular uptake flux within the cord tissue outside a thin strip of thickness h around each vessel (based on typical values for the thickness of the umbilical vessel walls [24]; table 1 and electronic supplementary material). The solute distribution and the corresponding integral fluxes (2.2) are computed via a finite-element solver of COMSOL Multiphysics${,}^{®}$ 6.0. We validated the use of the two-dimensional field C(X,Y) against direct computationally expensive solutions of the three-dimensional problem ((2.1); see electronic supplementary material, S5 for more details).

### 2.2. Image analysis of histology and ultrasound data

Histology and ultrasound images of human cord cross-sections were taken from published literature [3,18,26–31]. The histology data (stained with haematoxylin and eosin; figure 1b) corresponds to normal pregnancies, with gestational age varying from 37 to 42 weeks; ultrasound data included cords from the third trimester (see electronic supplementary material, table S4 for more details on data sources).

A semi-automated Python script (electronic supplementary material, S6) was used to extract structural metrics from the images, including effective radii and distances between each umbilical vessel, as well as the two characteristic angles (figure 1b). All measurements were normalized by the cord’s radius such that the scaled cord’s radius for each measurement is of unit length. The shape irregularity of each cord was quantified by the circularity parameter $4\pi A/(P{)}^{2}$, where A is the cross-sectional area and P is the perimeter of the cord.

## 3. Results

### 3.1. Cord coiling and vascular proximity promote solute and heat shunting

**Figure 2.**
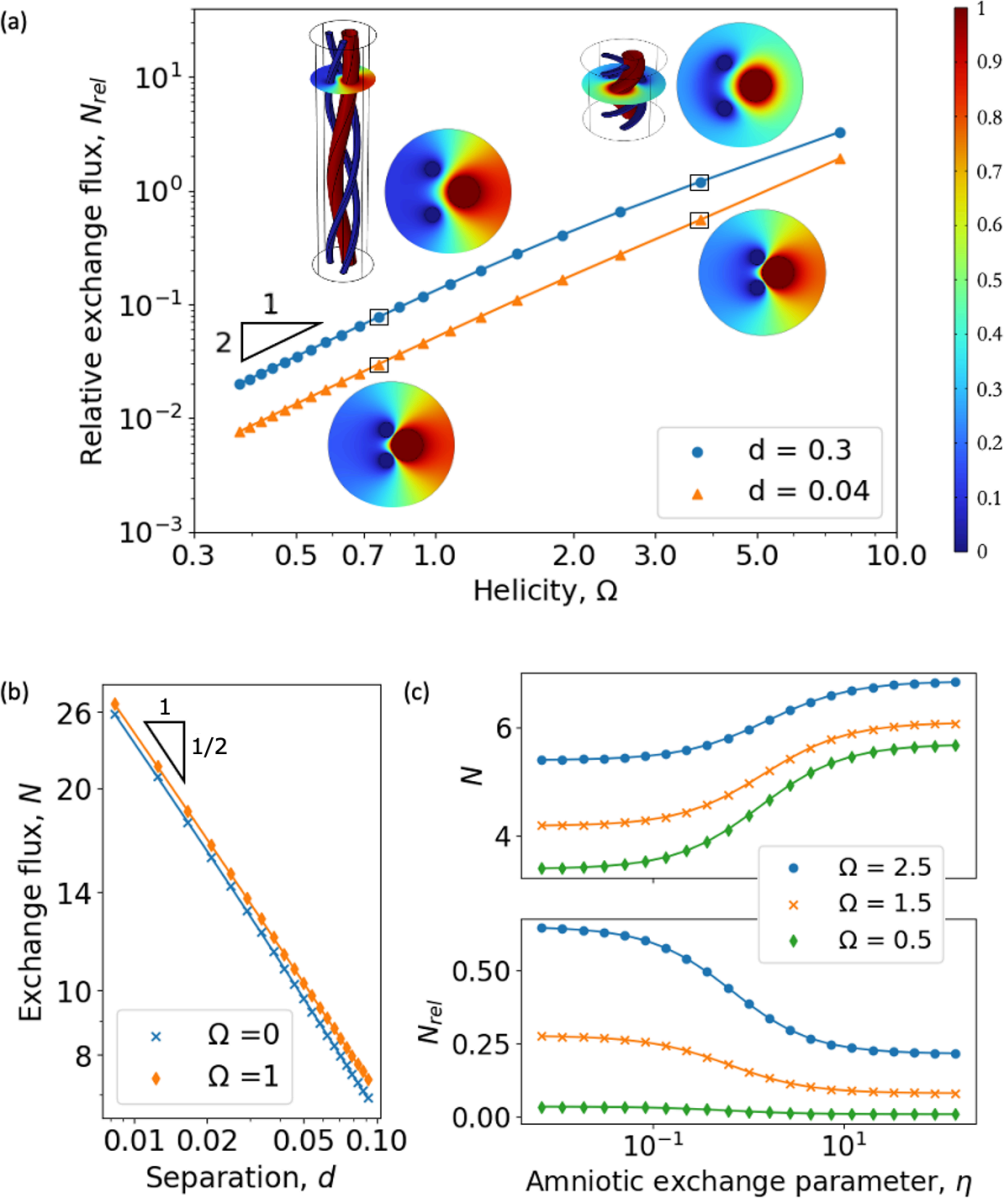
The impact of cord helicity and vascular proximity on solute exchange. (a) Relative exchange flux $N_{\mathrm{rel}}$ per unit length (3.1) of the UV versus cord helicity Ω for different UA–UV separation distances d=0.3 and d=0.04, with α=0,η=0. For weakly coiled cords, the solute (heat) exchange amplification scales as $\Omega^{2}$ (relative to the uncoiled vessels of the same length). Concentration fields for four different vessel configurations and three-dimensional diagrams show a normocoiled (Ω = 0.75) and hypercoiled (Ω = 3.77) cord, at parameters highlighted with squares on the graph. Cords with Ω<0.4 are hypocoiled, while cords with Ω>2 are hypercoiled. The colour bar shows solute concentration in normalized units (venous concentration is unity and arterial concentration is zero). (b) Computed exchange flux per unit length of the vein N for small separation d. In the limit of small d, the leading-order flux is $O(d^{-1/2})$. Here, α=0,η=0. (c) Exchange flux N and relative exchange flux $N_{\mathrm{rel}}$ for different values of the exchange parameter η in the cord boundary condition (2.1b ) for d=0.33. Other geometric parameters in (a–c) were fixed: $R_{v}=0.25$, $r_{v}=0.25$, $r_{a}=0.12$, $\theta_{1}=0.8\pi$, $\theta_{2}=1.2\pi$ (see electronic supplementary material for more details). The triangles in (a) and (b) show the relative fold-change in the variables.


We first explore how the structural parameters affect solute fluxes between the three vessels in a cord. Figure 2a illustrates the difference in exchange flux for helical (Ω>0) and straight (Ω=0) vessel geometries, in terms of the relative exchange flux

(3.1) $$\begin{array}{lcr} & N_{rel}=\frac{N(\Omega ,\alpha ,\beta ,\eta )-N(0,\alpha ,\beta ,\eta )}{N(0,\alpha ,\beta ,\eta )}, & \end{array}$$

assuming initially that α=η=0. Small Ω corresponds to weakly coiled cords, while large Ω corresponds to a tightly coiled cord. This is shown in the three-dimensional representations of one complete coil for Ω=0.75 and Ω=3.77 (figure 2a, insets). For large Ω, $N_{rel}$ exceeds unity, implying that the flux can be twice as large as the flux for straight vessels, because tightly coiled cords provide a larger internal surface area for exchange to take place. The concentration field for Ω=3.77, d=0.3 (figure 2a, inset, top right) varies weakly around the cord perimeter, showing how coiling promotes direct solute exchange between vessels. In contrast, the flux for weakly coiled vessels is similar to the flux for straight vessels. Figure 2a shows that the first correction to the straight vessel flux is of order $\Omega^{2}$. A weak helicity Ω≪1 approximation for flux can therefore be obtained by perturbing the three-dimensional problem (2.1a) in powers of $\Omega^{2}$.

Since in the problem for C(X,Y) the helicity term appears as $\Omega^{2}$, there is no evidence of chirality in the concentration fields in figure 2a. In the electronic supplementary material, figure S8b,c, we confirm that two configurations with opposite chirality have the same cross-sectional concentration fields. The position of the arteries in the configuration considered in figure 2a is symmetrical relative to the x-axis. This leads to the symmetry C(X,Y)=C(X,−Y). The electronic supplementary material, figure S8a, shows concentration fields with increasing helicity for asymmetric configurations. Breaking the symmetry introduces distortion in the concentration fields, but the trend in relative flux is the same as in figure 2a.

Another geometric parameter that contributes to the exchange of solutes is the separation distance d between vessels. The flux is larger for the umbilical vessels that are closer together because concentration gradients are steeper, as shown in figure 2a, colour map inserts. Indeed, figure 2b illustrates how, as d gets smaller, the flux in the gap between the vessels of $O(d^{-1/2})$ dominates over the flux outside the gap (see electronic supplementary material, S3 for more details), and the weak helicity correction of $O(d^{1/2}\;\Omega^{2})$ provides a good approximation (d=0.04; figure 2a). On the other hand, for larger d, the effect of helicity becomes more dominant, as demonstrated by the larger excess flux $N_{rel}$ (3.1) relative to the flux in the uncoiled geometry.

Figure 2c illustrates the effects of changing the amniotic exchange parameter η, which controls the outer boundary condition (2.1b), on the exchange flux. When η→0, the outer boundary is completely isolated, and the concentration gradients in the cord tissue are driven entirely by the interaction between the umbilical vessels, as in figure 2a,b. When η→∞, the flux increases since the additional sink, the amniotic fluid, forces steeper concentration gradients within the cord tissue. For weakly coiled cords, N almost doubles as η→∞. In contrast, the relative difference in exchange flux $N_{\mathrm{rel}}$ for different Ω shows that the role of helicity is strongest for η=0 ($N_{\mathrm{rel}}(\Omega =2.5)-N_{\mathrm{rel}}(\Omega =0.5)\approx 0.6$), compared with η→∞, when this difference falls ($N_{\mathrm{rel}}(\Omega =2.5)-N_{\mathrm{rel}}(\Omega =0.5)\approx 0.2$). We use the no-flux condition (η=0) for the remainder of this work because the effect of helicity is maximized, providing an upper bound for the relative diffusive coupling $N_{rel}$ between the umbilical vessels in this limit.

For the range of exchange fluxes N predicted in figure 2, the characteristic values of the Damköhler number (2.3), which quantifies the extent of shunting between the UV and UA, are ${\mathrm{Da}}_{{\text{O}}_{2}}∼10^{-5}$ and ${\mathrm{Da}}_{\text{heat}}∼10^{-1}$ for oxygen and heat, respectively (see electronic supplementary material, S8 for more details). Thus, although no physiological shunting is expected for oxygen exchange, heat exchange could be sensitive to the cord configuration.

### 3.2. Configurations yielding maximum and minimum exchange

**Figure 3.**
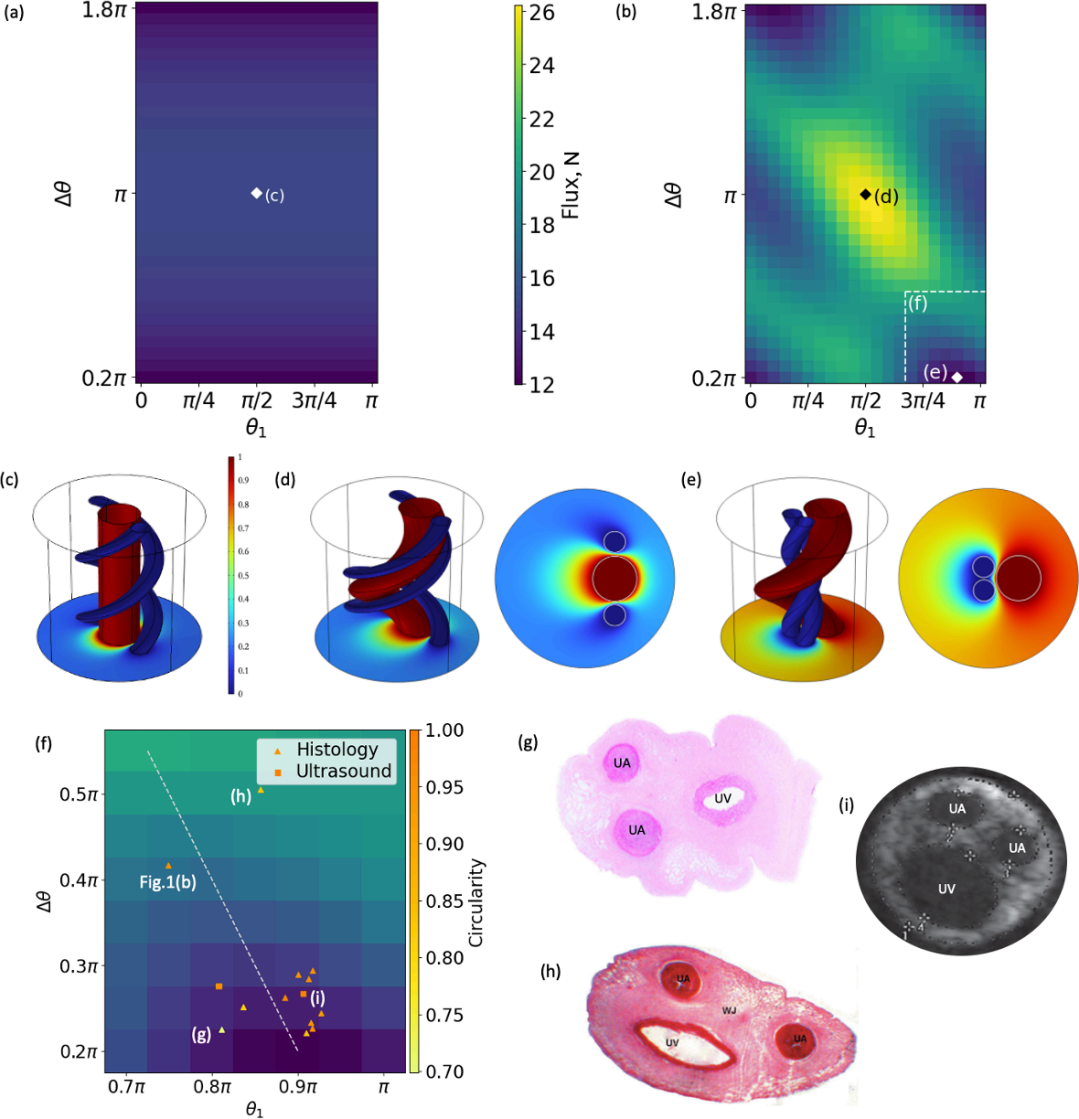
Solute exchange flux **N** for different cord configurations in the ($\theta_{1}$, Δθ) parameter space (figure 1b). The geometrical parameters used were $r_{v}=0.25,r_{a}=0.12,d=0.04,\eta =0,\Omega =3.77$ (table 1) for (a) $R_{v}=0$ (a straight vein, as shown in (c)) and (b) $R_{v}=0.21$. Cord configurations predicted by the computational model (e) ($\theta_{1}=0.9\pi$; Δθ=0.2π) that minimize and (d) ($\theta_{1}=0.5\pi$; Δθ=π) maximize solute exchange between the vessels (shown in (b)). Solute concentrations are plotted in normalized units, varying from C=0 on the arterial surfaces (blue) to C=1 on the surface of the vein (red). (f) Magnification of a region in (b) showing estimated angles obtained from histology and from ultrasound images of human cord cross-sections from healthy pregnancies. The line of symmetry (dashed) has a slope of −2 and a zero intercept at $\theta_{1}=\pi$. (g) and (h) are examples of histology images with irregular shapes, circularity less than 1 ([30], reproduced under CC BY 4.0, and [26], reproduced by permission of Taylor & Francis Ltd, tandfonline.com). (i) Ultrasound image of a cord with approximately unit circularity ([29], reproduced by permission of Karger Publishers, © 2009).

We now consider cross-sectional cord configurations with two arteries in different positions by changing the angles $\theta_{1}$ and $\Delta \theta =|\theta_{2}-\theta_{1}|$ (figure 1b). We keep the artery–vein distance (d=0.04) constant and use $\theta_{1}\in \left[0,\pi \right]$, while $\theta_{2}\in \left[0,2\pi \right)$. We plot the exchange flux N for all combinations of $\theta_{1}$ and $\theta_{2}$ for tightly coiled (Ω=3.77) configurations with a straight vein and zero offset $R_{v}=0$ (figure 3a,c) and for a coiled vein with an offset from the cord centreline $R_{v}=0.25$ (figure 3b,d). Configurations with overlapping arteries were excluded from the map; the smallest possible difference in angles such that the arteries do not intersect was Δθ=0.2π.

When the vein is located at the centre of the cord ($R_{v}=0$; figure 3c), the exchange flux is invariant with respect to $\theta_{1}$ and depends only on the relative position Δθ of the two UAs around the UV (figure 3a). The exchange is maximized at Δθ=π when the arteries are aligned on opposite sides of the vein (figure 3c). Increasing the offset (helical radius) of the vein from the cord’s centre ($R_{v}>0$), and hence twisting the vein, amplifies the solute flux (the maximum N increases from about 15 to 26; figure 3b). Furthermore, the displaced vein, which acts as a source, breaks the radial symmetry of the concentration field and produces a complex landscape of enhanced and reduced values for the exchange flux, as shown in figure 3b. The region around the global maximum ($\theta_{1}\approx 0.5\pi$ and Δθ≈π) corresponds to the configuration where the arteries are aligned on either side of the vein, perpendicular to the x-axis that passes through the centre of the cord and centre of the vein (figure 3d). Similarly, local maxima are attained when the arteries are close to each other in the region $\theta_{1}\in \left[\pi /4,\pi /2\right]$. The global minimum ($\theta_{1}\approx 0.9\pi$ and Δθ≈0.2π) is achieved when arteries are close to each other and are placed along the x-axis near the centre of the cord (figure 3e).

The configurations that maximize the exchange flux in both scenarios (figure 3c,d) are associated with UAs that straddle the UV, allowing for a larger surface area. However, a more general map in figure 3b reveals that the exchange flux is smaller when the two UAs are aligned close to the x-axis ($\theta_{1}\approx \pi$; figure 3e) than when they are aligned orthogonally to it ($\theta_{1}\approx \pi /2$). This indicates the importance of a specific three-dimensional arrangement of the cord’s vessels, which goes beyond the effective surface area of the UA–UV interface.

To determine realistic configurations of the human umbilical cord, we estimated the angles $\theta_{1}$ and Δθ from histological studies and ultrasound images of umbilical cords from normal pregnancies. Figure 3g,h shows examples of histological images of the human umbilical cord that highlight the irregular, non-circular shape of the cross-section. Shortly after birth, the UAs undergo constriction in order to prevent fetal blood loss. This effect is seen in the histological images, where the thick arterial walls close off the lumen. Similarly, the lumen of the UV collapses due to changes in pressure and presents an elliptical cross-section. In contrast, the ultrasound image in figure 3i shows that an *in vivo* cord has a more regular circular cross-section. The lumen of the vessels is also more visible and has a circular shape. This supports the model assumption of circular cross-sections for both the cord and the vessels, since it better represents *in utero* conditions.

Figure 3f shows these measurements plotted in the context of the exchange flux map (figure 3b). The observed vascular configurations approximately lie on the line of symmetry $\Delta \theta =-2\theta_{1}+2\pi$ for which the position of the arteries is symmetrical with respect to the x-axis, passing through the centre of the cord and the centre of the vein. The model indicates that this approximately symmetric arrangement of the UAs, with a small Δθ, minimizes the exchange flux in the 14 human cords from healthy pregnancies that we investigated.

### 3.3. ‘Virtual’ *vasa vasorum*

**Figure 4.**
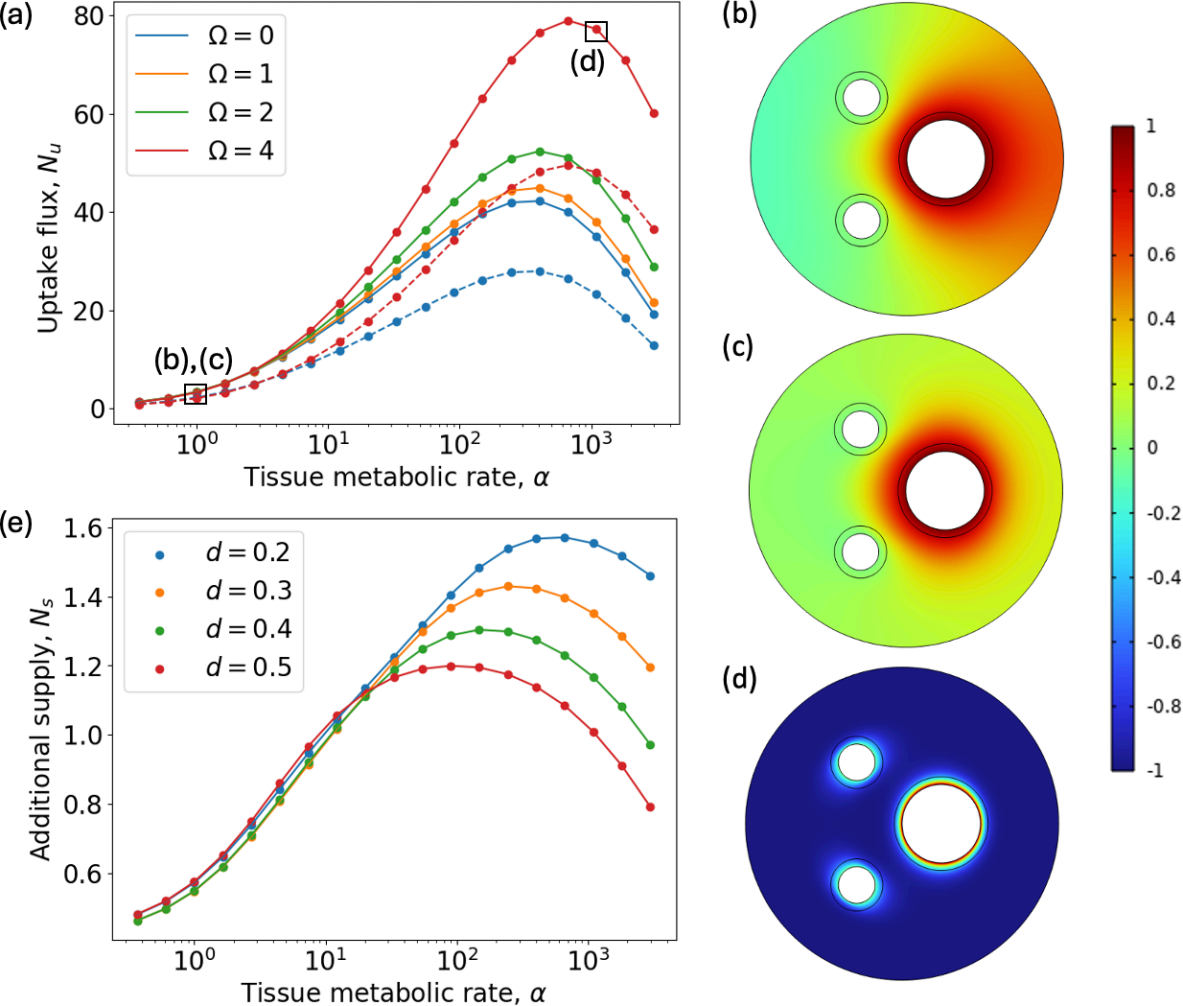
The role of umbilical vascular structure in the cord tissue oxygenation. (a) Uptake flux $N_{u}$ (2.2) by the cord tissue (excluding a thin strip of thickness h=0.05 outside the vascular lumens), for varying rate of tissue metabolism α and cord helicity Ω. The solid lines correspond to β=1, while the dotted lines show β=2. Scaled concentration fields for (b) Ω=0;α=1, (c) Ω=4;α=1 and (d) $\Omega =4;\alpha =10^{3}$ plotted in normalized units. (e) The contribution of the UV to the cord tissue oxygenation, quantified by the uptake flux $N_{s}$ relative to the flux in the case of diffusive oxygen supply by the UAs alone. Colours indicate different inter-vessel distances d. Other geometrical parameters used are as follows: $R_{v}=0.25$, $r_{v}=0.25$, $r_{a}=0.12$, d=0.3, $\theta_{1}=0.8\pi$, $\theta_{2}=1.2\pi$ and η=0.

We hypothesize that the enhanced exchange between helical vessels might boost the supply of oxygen to the walls of the UAs and hence function as a ‘virtual’ *vasa vasorum*. In figure 4a, we show the effects of helicity Ω, and the relative solute concentration difference between the UA and the UV β, on the extravascular uptake flux $N_{u}$ (see (2.2)). Similarly to figure 2, the uptake flux increases with coiling; however, for weak tissue metabolism, the effect is less noticeable. Figure 4b,c shows similar effects to figure 2a, where concentration fields become more uniform as Ω increases. For a higher metabolic rate α, the impact of helicity gets stronger, and the distortions in the concentration fields in the cord tissue also have a stronger impact on the uptake flux. Figure 4d shows boundary layers around the arteries, which have an elliptical shape due to the effects of helicity on the concentration field. Additionally, the value of α for which the maximum flux is attained increases for larger Ω.

Figure 4a shows a non-monotonic increase in the extravascular uptake flux $N_{u}$ (2.2) as the metabolic rate parameter increases. Clearly, for α=0, the uptake flux in the cord tissue must be zero. Large α leads to significant spatial gradients of concentration localized in narrow regions around the vessels (figure 4d), reducing solute exchange between the UA and the UV. We assess the impact of metabolism on the oxygen supply to the avascular cord tissue by excluding thin strips of size h (comparable to the vessel wall thickness). Therefore, the uptake flux $N_{u}$ (figure 4a) tends to zero for sufficiently strong metabolism ($\alpha \gg 1/h^{2}$), when the tissue oxygenation outside the immediate vicinity of the vascular lumen is negligible. Figure 4a also illustrates the role of the relative venous–arterial concentration difference β for the baseline case of β=1 (oxygen concentration in the UV is twice that of the UA) and for β=2 (the UV concentration is three times that of the UA). The apparent drop in peak $N_{u}$ at higher β masks an increase in the absolute uptake flux that scales with this concentration difference $C_{v}-C_{a}$ (in dimensional variables; see (2.2)).

To explore the contribution of the UV to cord tissue oxygenation more directly, we consider a base case in which oxygen in the cord tissue is supplied only by the UA. We compare the net uptake flux $N_{u}$ with this base uptake flux $N_{b}$ by calculating $N_{s}=(N_{u}-N_{b})/N_{b}$, which quantifies the additional oxygen supplied to the tissue due to the presence of the UV (see electronic supplementary material, S4 for more details). Figure 4e shows how $N_{s}$ varies with α for different vessel separations d. Similarly to figure 4a, there is a non-monotonic increase in excess oxygen supply. As vessels get closer to each other, a large α similarly blocks the interactions between the umbilical vessels and hence diminishes the exchange between them. However, when the vessel separation d is small, the additional oxygen supply to the cord tissue is almost twice as large compared with the base case (figure 4e).

## 4. Discussion

Beyond its obvious role as a conduit for blood enriched with oxygen and nutrients between the placenta and the fetus, rather little is known about the structure–function relationship of the umbilical cord in pregnancy. While functional observations in the cord *in vivo* are typically limited to ultrasound measurements of blood flow, computational modelling [13,15] could offer deeper insight into the physiological role of the umbilical cord.

In this study, we examine the hypothesis that there exists a functional shunt between the umbilical vessels through a mathematical model that describes solute diffusion and uptake in the cord tissue. By exploiting a natural transformation of the coordinate system, we encapsulate the complex three-dimensional helical geometry of the umbilical vessels in a computationally efficient and interpretable two-dimensional problem, which is formulated in the cross-sectional plane of the cord. The model reveals that cord coiling and vascular proximity promote solute exchange between the umbilical vessels (figure 2). In the limit of moderate helicity (Ω≤1), the model shows that the exchange flux is approximately proportional to the square of the cord helicity, or, equivalently, the UCI augmented by the cord diameter D: $\Omega^{2}∼D^{2}(\text{UCI}{)}^{2}$. Similarly, in the limit of a thin gap between the umbilical vessels, separated by the relative distance d≪1, the model predicts that the dominant contribution to the exchange flux is $O(d^{-1/2})$. Similar to the earlier results for blood flow modelling in the umbilical cord [13,32], these geometric determinants show that the UCI alone does not capture the essential features of solute exchange and thus can be a poor indicator of clinical pathology.

The simulations also reveal a non-trivial relationship between the vascular configurations in the cross-section of the umbilical cord and the extent of solute exchange between the vessels (figure 3). We use theoretical predictions to interpret the structural images of cords from healthy pregnancies. In all the samples we investigated, the UAs were positioned approximately symmetrically with respect to the axis, passing through the cord centre and the centre of the vein, so that the observed configurations tend to minimize shunting between the umbilical vessels. To put this in the context of the typical transported solutes, we assessed the flux exchanged between the umbilical vessels relative to the flux delivered to the fetus by the UV, as quantified by a Damköhler number Da (see (2.3)). Oxygen transport in the umbilical cord is diffusion-limited (advection-dominated, as represented by a small Da), implying that the amount of oxygen exchanged between the cord vessels can be neglected. In contrast, the diffusive coupling for heat transfer may be significant, because the relevant Da is significantly larger. Given the tendency to minimize this coupling in the measured cord configurations, it opens an intriguing possibility that the cord structure can be partially explained by the demands of fetal thermoregulation.

Although the human umbilical cord tissue lacks *vasa vasorum* [33], the cord’s WJ contains mesenchymal stem cells (MSCs), fibroblasts and other connective tissue cells [18], all of which are metabolically active and depend on oxygen to perform their functions, which are essential for the health of the cord (such as tissue repair by the MSCs and collagen synthesis by the fibroblasts). Our model demonstrates how the functional shunt between the umbilical vessels can act as ‘virtual’ *vasa vasorum* by amplifying the oxygen supply to the cord tissue. While the cells residing in the WJ are probably adapted to a relatively low oxygen environment compared with other tissues [33], we hypothesize that abnormal coiling and vascular separation distance could adversely affect the development and function of the perivascular cord tissue; however, further experimental studies are needed to evaluate the physiological significance of this mechanism.

Our modelling approach has many limitations and assumptions that warrant further study. For example, we neglected the effects of potential solute heterogeneity on the surface of umbilical vessels (which is important in parallel uncoiled exchanger systems [34]). However, the length of the cord and the helicity-induced secondary flows [35,36] will promote the mixing of solutes in umbilical vessels, justifying to some extent the azimuthally uniform approximation. We also neglected axial concentration gradients and assumed a constant coiling pitch, whereas human cords can exhibit slow pitch variations [13]. Future work can generalize this model by considering non-zero axial concentration gradients to study exchange along the whole cord and explore more accurately how the functional shunt affects the transport of solutes between the placenta and the fetus.

Additionally, in this study, we primarily analysed the cord structure based on histological images; however, the *in vivo* structure of the cord is prone to significant perturbations post-delivery. For example, the UV lumen collapses due to changes in pressure, while the UA lumen contracts, and the clots within it help avoid neonatal blood loss [37]. These effects can be seen in figure 3g,h, where the vein has an elliptical shape, the arterial lumens are not readily visible and the cord’s cross-section can have an irregular (non-circular) shape. In contrast, ultrasound images better reproduce the *in utero* environment since the vessels are not collapsed or constricted, with more circular shapes and visible lumens (figure 3i). Our analysis relies on the relative centre-of-mass locations of the umbilical vessels, rather than finer shape details, making our results less sensitive to these *ex vivo* distortions. Nevertheless, in future studies, ultrasound images should be used for a more accurate representation of the umbilical vascular structure.

We have also limited our analysis to cords from normal human pregnancies. Pathological studies often report thin cords with a significant decrease in WJ [38], and instances of cords with a single UA, which have been associated with FGR, pre-eclampsia and other adverse pregnancy outcomes [39]. Similarly, the morphology of the umbilical cord is known to vary significantly across different placental mammals [19]. These structural differences motivate the need for a systematic quantitative comparison of the cord’s performance in healthy and pathological human pregnancies (using multiple imaging modalities [40]), as well as its interspecies variations [41,42].

To conclude, our study offers a new perspective on the structure–function relationship of the umbilical cord by investigating solute exchange between umbilical vessels. Using image-based mathematical modelling, we have demonstrated how cord coiling can significantly enhance diffusive coupling between vessels, while the relative vascular arrangement in human cords typically minimizes this coupling. These findings have potential implications for the thermal regulation of the fetus and the oxygenation of the cord tissue. The approach outlined in this paper could be expanded to address the impact of this functional shunt on fetal development in human pregnancy pathologies and across different species.

## Data Availability

All data needed to evaluate the conclusions in the paper are present in the paper and/or the electronic supplementary materials. The associated structural datasets and computational codes can be accessed via the Figshare repository [43]. Supplementary material is available online [44].

## References

[1] Tantbirojn P, Saleemuddin A, Sirois K, Crum CP, Boyd TK, Tworoger S, Parast MM. 2009 Gross abnormalities of the umbilical cord: related placental histology and clinical significance. Placenta **30**, 1083–1088. (10.1016/j.placenta.2009.09.005) 19853300

[2] Makarchuk D. 2023 Pregnancy and umbilical cord pathology: structural and functional parameters of the umbilical cord. J. Med. **16**, 1282–1291. (10.25122/jml-2023-0025) PMC10652671 38024812

[3] Blanco MV, Vega HR, Guerri-Guttenberg RA, Giuliano R, Grana DR, Azzato F, Milei J. 2011 Histopathology and histomorphometry of umbilical cord blood vessels. Findings in normal and high risk pregnancies. Artery Res. **5**, 50. (10.1016/j.artres.2011.02.001)

[4] Strong TH, Jarles DL, Vega JS, Feldman DB. 1994 The umbilical coiling index. Am. J. Obstet. Gynecol. **170**, 29–32. (10.1016/s0002-9378(13)70274-6) 8296839

[5] van Dijk CC, Franx A, de Laat MWM, Bruinse HW, Visser GHA, Nikkels PGJ. 2002 The umbilical coiling index in normal pregnancy. J. Matern. Fetal Neonatal Med. **11**, 280–283. (10.1080/jmf.11.4.280.283) 12375686

[6] Machin GA, Ackerman J, Gilbert-Barness E. 2000 Abnormal umbilical cord coiling is associated with adverse perinatal outcomes. Pediatr. Dev. Pathol. **3**, 462–471. (10.1007/s100240010103) 10890931

[7] Strong TH, Elliott JP, Radin TG. 1993 Non-coiled umbilical blood vessels: a new marker for the fetus at risk. Obstet. Gynecol. **81**, 409–411. 8437796

[8] Jensen OE, Chernyavsky IL. 2019 Blood flow and transport in the human placenta. Annu. Rev. Fluid Mech. **51**, 25–47. (10.1146/annurev-fluid-010518-040219) 38410641 PMC7615669

[9] Clark AR, Lin M, Tawhai M, Saghian R, James JL. 2015 Multiscale modelling of the feto–placental vasculature. Interface Focus **5**, 20140078. (10.1098/rsfs.2014.0078) 25844150 PMC4342946

[10] Pearce P, Brownbill P, Janáček J, Jirkovská M, Kubínová L, Chernyavsky IL, Jensen OE. 2016 Image-based modeling of blood flow and oxygen transfer in feto-placental capillaries. PLoS One **11**, e0165369. (10.1371/journal.pone.0165369) 27788214 PMC5082864

[11] Plitman Mayo R, Charnock-Jones DS, Burton GJ, Oyen ML. 2016 Three-dimensional modeling of human placental terminal villi. Placenta **43**, 54–60. (10.1016/j.placenta.2016.05.001) 27324100

[12] Waters SL, Guiot C. 2001 Flow in an elastic tube subject to prescribed forcing: a model of umbilical venous flow. J. Theor. Med. **3**, 287–298. (10.1080/10273660108833081)

[13] Wilke DJ, Denier JP, Khong TY, Mattner TW. 2018 Pressure and flow in the umbilical cord. J. Biomech. **79**, 78–87. (10.1016/j.jbiomech.2018.07.044) 30146174

[14] Kaplan AD, Jaffa AJ, Timor IE, Elad D. 2010 Hemodynamic analysis of arterial blood flow in the coiled umbilical cord. Reprod. Sci. **17**, 258–268. (10.1177/1933719109351596) 20023275

[15] Saw SN, Dawn C, Biswas A, Mattar CNZ, Yap CH. 2017 Characterization of the in vivo wall shear stress environment of human fetus umbilical arteries and veins. Biomech. Model. Mechanobiol. **16**, 197–211. (10.1007/s10237-016-0810-5) 27456489

[16] Syed S, O’Sullivan TL, Phillips KP. 2022 Extreme heat and pregnancy outcomes: a scoping review of the epidemiological evidence. Int. J. Environ. Res. Public Health **19**, 2412. (10.3390/ijerph19042412) 35206601 PMC8874707

[17] Kasiteropoulou D, Topalidou A, Downe S. 2020 A computational fluid dynamics modelling of maternal-fetal heat exchange and blood flow in the umbilical cord. PLoS One **15**, e0231997. (10.1371/journal.pone.0231997) 32722669 PMC7386597

[18] Benirschke B, Burton GJ, Baergen RN. 2012 Pathology of the human placenta. Berlin, Germany: Springer. (10.1007/978-3-642-23941-0)

[19] Benirschke K. 2007 Comparative placentation. See http://placentation.ucsd.edu.

[20] Rubin JM, Fowlkes JB, Pinter SZ, Treadwell MC, Kripfgans OD. 2022 Umbilical vein pulse wave spectral analysis: a possible method for placental assessment through evaluation of maternal and fetal flow components. J. Ultrasound Med. **41**, 2445–2457. (10.1002/jum.15927) 34935157 PMC10204125

[21] de Laat M, Franx A, van Alderen E, Nikkels P, Visser G. 2005 The umbilical coiling index, a review of the literature. J. Matern. Fetal Neonatal Med. **17**, 93–100. (10.1080/jmf.17.2.93.100) 16076615

[22] Weissman A, Jakobi P, Bronshtein M, Goldstein I. 1994 Sonographic measurements of the umbilical cord and vessels during normal pregnancies. J. Ultrasound Med. **13**, 11–14. (10.7863/jum.1994.13.1.11) 7636947

[23] Raio L, Ghezzi F, Di Naro E, Gomez R, Franchi M, Mazor M, Brühwiler H. 1999 Sonographic measurement of the umbilical cord and fetal anthropometric parameters. Eur. J. Obstet. Gynecol. Reprod. Biol. **83**, 131–135. (10.1016/s0301-2115(98)00314-5) 10391521

[24] Gayatri R, Crasta J, Thomas T, Pratibha D, Thomas A, Sridhar TS, Kurpad AV. 2017 Structural analysis of the umbilical cord and its vessels in intrauterine growth restriction and pre-eclampsia. J. Fetal Med. **04**, 85–92. (10.1007/s40556-017-0118-2)

[25] Tuan-Mu HY, Chang YH, Hu JJ. 2020 Removal of an abluminal lining improves decellularization of human umbilical arteries. Sci. Rep. **10**, 10556. (10.1038/s41598-020-67417-4) 32601366 PMC7324607

[26] Thomas MR, Bhatia JK, Kumar S, Boruah D. 2020 The histology and histomorphometry of umbilical cord cross section in preeclampsia and normal pregnancies: a comparative study. J. Histotechnol. **43**, 109–117. (10.1080/01478885.2020.1734741) 32160831

[27] Herzog EM *et al*. 2017 Impact of early- and late-onset preeclampsia on features of placental and newborn vascular health. Placenta **49**, 72–79. (10.1016/j.placenta.2016.11.014) 28012458

[28] Blanco-Elices C, Chato-Astrain J, González-González A, Sánchez-Porras D, Carriel V, Fernández-Valadés R, Sánchez-Quevedo M del C, Alaminos M, Garzón I. 2022 Histological profiling of the human umbilical cord: a potential alternative cell source in tissue engineering. J. Pers. Med. **12**, 648. (10.3390/jpm12040648) 35455764 PMC9028794

[29] Kurita M, Hasegawa J, Mikoshiba T, Purwosunu Y, Matsuoka R, Ichizuka K, Sekizawa A, Okai T. 2009 Ultrasound evaluation of the amount of Wharton’s jelly and the umbilical coiling index. Fetal Diagn. Ther. **26**, 85–89. (10.1159/000238118) 19752523

[30] Kurakazu M, Kurakazu M, Murata M, Miyamoto T, Takahashi Y, Hamasaki M, Ohta E, Yotsumoto F, Miyamoto S. 2019 A partial supernumerary umbilical vein: a case report. J. Med. Case Rep. **13**, 149. (10.1186/s13256-019-2094-8) 31101065 PMC6525366

[31] Skheel Al-Jebory HD, Bander Asaad U. 2016 Cross sectional area of umbilical cord as a predictor for neonatal birth weight. Mustansiriya Med. J. **15**, 46. (10.4103/2070-1128.247890)

[32] Wilke DJ, Denier JP, Khong TY, Mattner TW. 2021 Estimating umbilical cord flow resistance from measurements of the whole cord. Placenta **103**, 180–187. (10.1016/j.placenta.2020.09.066) 33160251

[33] Davies JE, Walker JT, Keating A. 2017 Concise review: Wharton’s jelly: the rich, but enigmatic, source of mesenchymal stromal cells. Stem Cells Transl. Med. **6**, 1620–1630. (10.1002/sctm.16-0492) 28488282 PMC5689772

[34] Pierre C, Bouyssier J, de Gournay F, Plouraboué F. 2014 Numerical computation of 3D heat transfer in complex parallel heat exchangers using generalized Graetz modes. J. Comput. Phys. **268**, 84–105. (10.1016/j.jcp.2014.02.037)

[35] Dean WR. 1928 LXXII. The stream-line motion of fluid in a curved pipe (second paper). Phil. Mag. J. Sci. **5**, 673–695. (10.1080/14786440408564513)

[36] Zabielski L, Mestel AJ. 1998 Steady flow in a helically symmetric pipe. J. Fluid Mech. **370**, 297–320. (10.1017/s0022112098002006)

[37] Nandadasa S *et al*. 2020 Vascular dimorphism ensured by regulated proteoglycan dynamics favors rapid umbilical artery closure at birth. eLife **9**, e60683. (10.7554/elife.60683) 32909945 PMC7529456

[38] Debebe SK *et al*. 2020 Wharton’s jelly area and its association with placental morphometry and pathology. Placenta **94**, 34–38. (10.1016/j.placenta.2020.03.008) 32421532 PMC7491570

[39] Bruch JF, Sibony O, Benali K, Challier JC, Blot P, Nessmann C. 1997 Computerized microscope morphometry of umbilical vessels from pregnancies with intrauterine growth retardation and abnormal umbilical artery Doppler. Hum. Pathol. **28**, 1139–1145. (10.1016/s0046-8177(97)90251-3) 9343320

[40] Clark AR, Lee TC, James JL. 2021 Computational modeling of the interactions between the maternal and fetal circulations in human pregnancy. WIREs Mech. Dis. **13**, e1502. (10.1002/wsbm.1502) 32744412

[41] Laundon D, Gostling NJ, Sengers BG, Chavatte-Palmer P, Lewis RM. 2024 Placental evolution from a three-dimensional and multiscale structural perspective. Evolution **78**, 13–25. (10.1093/evolut/qpad209) 37974468

[42] Bappoo N *et al*. 2024 Feto-placental vascular structure and in silico haemodynamics: of mice, rats, and human. Placenta **158**, 175–184. (10.1016/j.placenta.2024.10.020) 39476476

[43] Wan T, Johnstone ED, Saw SN, Jensen OE, Chernyavsky IL. 2025 Supplementary datasets and codes from: A functional shunt in the umbilical cord: the role of coiling in solute and heat transfer. Figshare (10.6084/m9.figshare.28382012)40987331

[44] Wan T, Johnstone ED, Saw SN, Jensen OE, Chernyavsky IL. 2025 Supplementary material from: A functional shunt in the umbilical cord: the role of coiling in solute and heat transfer. Figshare. (10.6084/m9.figshare.c.8026204)40987331

